# Soft crystal lattice and large anharmonicity facilitate the self-trapped excitonic emission in ultrathin 2D nanoplates of RbPb_2_Br_5_†

**DOI:** 10.1039/d2sc02992h

**Published:** 2022-08-01

**Authors:** Jayita Pradhan, Anustoop Das, Kaushik Kundu, Chahat, Kanishka Biswas

**Affiliations:** New Chemistry Unit, Jawaharlal Nehru Centre for Advanced Scientific Research (JNCASR) Jakkur P. O. Bangalore 560064 India kanishka@jncasr.ac.in; School of Advanced Materials, International Centre for Materials Science, Jawaharlal Nehru Centre for Advanced Scientific Research (JNCASR) Jakkur P. O. Bangalore 560064 India

## Abstract

Self-trapping of excitons (STE) and concomitant useful broadband emission in low-dimensional metal halides occur due to strong electron–phonon coupling, which exhibit potential applications in optoelectronics and solid-state lighting. Lattice softness and high anharmonicity in the low-dimensional structure can lead to transient structural distortion upon photoexcitation that should promote the spatial localization or trapping of charge carriers, which is essential for STE. Herein, we report the ligand-assisted reprecipitation synthesis of ultrathin (∼3.5 nm) two-dimensional (2D) metal halide, RbPb_2_Br_5_ nanoplates (NPLs), which demonstrate highly Stokes shifted and broadband emission covering most parts of the visible to near IR range (500–850 nm) with a long-lived photoluminescence (PL) lifetime. The excitation wavelength independent emission and emission wavelength independent excitation spectra along with the analogous PL decay kinetics of bulk and NPLs suggest the intrinsic nature of broadband emission. The experimental low sound velocity (∼1090 m s^−1^) and associated low bulk and shear moduli (10.10 and 5.51 GPa, respectively) indicate the large anharmonicity and significantly soft lattice structure, which trigger the broadband STE emission in 2D NPLs of RbPb_2_Br_5_. Strong electron-longitudinal optical (LO) phonon coupling results in broadband STE emission in 2D RbPb_2_Br_5_ NPLs.

## Abstract 2

The presence of large anharmonicity and lattice softness in low dimensional metal halide, RbPb_2_Br_5_ nanoplates, trigger strong electron–phonon coupling and consequently highly Stokes shifted broadband emission originates from self-trapped excitons.

## Graphical abstract

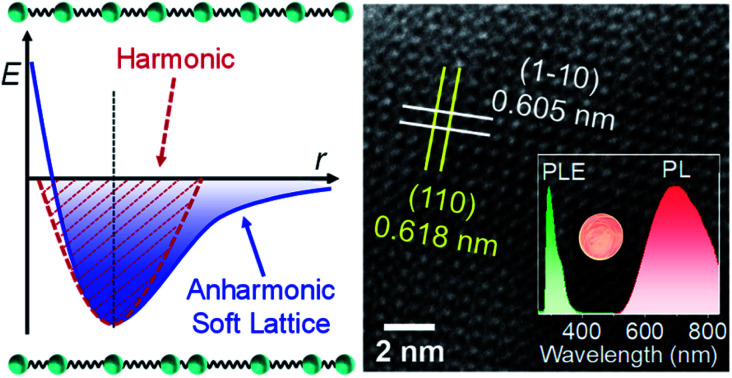

## Introduction

Metal halides have fascinated researchers due to their countless stimulating properties and potential applications in optoelectronics, photodetection and photovoltaics.^1–4^ The ease of their colloidal synthesis in the form of intensely luminescent nanocrystals along with their tuneable optoelectronic properties has attracted researchers from diverse fields.^3–6^ Recently, low-dimensional all-inorganic metal halides with two-dimensional (2D) layered structures such as APb_2_X_5_ (*e.g.* A = Cs; X = Cl, Br, I) have been examined as a noteworthy case.^7–9^ Among them, CsPb_2_Br_5_ with a layered structure turned into an emergent material, in which a Cs cation is sandwiched between [Pb_2_Br_5_]^−^ polyhedra,^9^ and exhibited high photoluminescence quantum yield (PLQY), colour tunability, and enhanced stability in humid environments and at high temperatures.^7,8^ However, the limited compositional variability of AB_2_X_5_ halide nanostructures is inadequate for exploring their optoelectronic properties in detail. Only a few potential alternatives are available to replace Cs in the all-inorganic AB_2_X_5_-type structures.^10,11^ Rb, as a neighbouring alkali metal of Cs, is suitable to form AB_2_X_5_-type metal halides. However, layered RbPb_2_Br_5_ has rarely been investigated except for a few electronic structural studies on its single crystals.^11,12^ Simple synthesis and fundamental understanding of the optical properties of RbPb_2_Br_5_ in the form of 2D nanoplates/nanosheets or nanocrystals are still elusive.

Dimensionality reduction facilitates the disconnection of the metal halide octahedra and the lattice becomes progressively softer. In such systems, self-trapped excitons (STEs) form due to local structural distortion upon photoexcitation.^13^ Strong electron–phonon coupling causes elastic lattice deformation and consequent STE results in broadband emission with a large Stokes shift, which showed promise for solid state lighting.^14^ Thereby, it would be exciting to study the optical properties and electron–phonon coupling in 2D layered RbPb_2_Br_5_ both in the form of bulk and ultrathin nanostructures.

Herein, we present simple ligand-assisted reprecipitation (LARP) synthesis of 2D ultrathin few-layer nanoplates (NPLs) of layered metal halide, RbPb_2_Br_5_, which displays broadband self-trapped excitonic (STE) emission. We have further synthesized controlled bulk polycrystals of RbPb_2_Br_5_ by solvent free mechanochemistry. Photoluminescence excitation (PLE) and PL spectra of RbPb_2_Br_5_ NPLs and bulk polycrystals are independent of emission and excitation wavelengths, which reveal that broadband emission originates from relaxation of the same excited state. We have observed a significant Stokes shifted (∼400 nm) emission with a long PL lifetime in the microsecond (μs) range. The temperature-dependent (15–350 K) PL studies confirm the strong electron-longitudinal optical (LO) phonon coupling in 2D RbPb_2_Br_5_ NPLs. Large lattice anharmonicity and low sound velocity (∼1090 m s^−1^) indicate the structure to be elastically very soft with significantly low bulk and shear moduli. The soft crystal lattice and strong electron–phonon coupling trigger the broadband STE emission in 2D NPLs of RbPb_2_Br_5_.

## Results and discussion

We have synthesized nanoplates (NPLs) of RbPb_2_Br_5_*via* the ligand-assisted reprecipitation (LARP) method at room temperature (Scheme S1a, ESI†). Typically, a dimethyl sulfoxide (DMSO) solution consisting of RbBr and PbBr_2_ at a stoichiometric molar ratio (1 : 2) along with organic ligands [*e.g.*, oleic acid (OA) and oleyamine (OLAm)] was swiftly injected into chloroform under vigorous stirring. After 20 s, the as-synthesized product was collected and washed with chloroform for further measurement. Further, we have synthesized micrometre sized particles (∼1 g) of RbPb_2_Br_5_ by implementing solid-state mechanochemical grinding with appropriate stoichiometric ratios of RbBr and PbBr_2_ (Scheme S1b, ESI†).

**Fig. 1:**
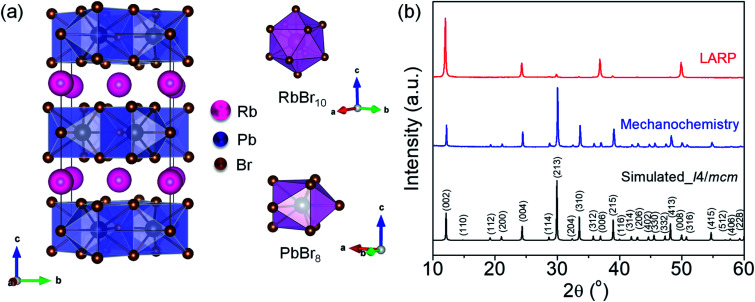
(a) Crystal structure of RbPb_2_Br_5_ viewed along the crystallographic *a*-axis and in the coordination environment of Rb and Pb. (b) PXRD patterns of RbPb_2_Br_5_, obtained by mechanochemistry and ligand-assisted reprecipitation (LARP) methods.

RbPb_2_Br_5_ adopts a tetragonal crystal structure with the *I*4/*mcm* space group, which consists of intercalated Rb^+^ ions sandwiched in between the 2D layers of [Pb_2_Br_5_]^−^ polyhedral units (Fig. 1a). In the tetragonal unit cell, the RbBr_10_ polyhedron exhibits a bicapped square antiprism unit, while the PbBr_8_ polyhedron shows a bicapped trigonal prism coordination (Fig. 1a).^15^ The powder X-ray diffraction (PXRD) patterns of the as-synthesized bulk and NPL samples have been indexed to the tetragonal phase of RbPb_2_Br_5_ (space group *I*4/*mcm*) (Fig. 1b).^15^ The PXRD pattern of the as-synthesized NPLs obtained from the LARP route shows diffraction peaks corresponding to mainly (002), (004), (006) and (008) planes, which clearly indicates the 2D growth along the <110> direction. The characteristic layered peak appears at 2*θ* = 11.66° corresponding to the (002) plane in both RbPb_2_Br_5_ bulk polycrystals and NPLs. We have studied the environmental and the thermal stabilities of the mechanochemically synthesized bulk RbPb_2_Br_5_ powders. The bulk polycrystals display excellent crystallinity and stability even after 250 days of being kept under ambient conditions (Fig. S1a, ESI†), whereas thermogravimetric analysis (TGA) reveals good thermal stability up to 465 °C (Fig. S1b, ESI†). Additionally, we have checked the thermal stability of the NPLs which reveals an analogous stability profile to that of the bulk polycrystals (Fig. S1c, ESI†). In the case of NPLs, we noticed an early weight loss of ∼2% between 25 °C and 235 °C (inset, Fig. S1c, ESI†). This is attributed to the removal of surface capping organic ligands (oleic acid and oleylamine).^16,17^

We have studied the PXRD patterns of the as-synthesized RbPb_2_Br_5_*via* the LARP method with and without the addition of capping ligands (OA and OLAm), and the comparison of the PXRD patterns reveals that the directional growth takes place only in the presence of OA and OLAm (Fig. S2, ESI†). The presence of capping ligands has been further verified by Fourier transform infrared (FTIR) spectroscopy (Fig. S3, ESI†). The FTIR spectrum of the as-synthesized NPLs exhibits the symmetric and asymmetric stretching vibrations of –CH_2_ and –CH_3_ moieties in the range of 2850–2950 cm^−1^, which can be assigned to the oleyl group [*i.e.*, CH_3_(CH_2_)_7_–CH=CH–(CH_2_)_8_^−^] from both OA and OLAm.^18,19^ The –CH_2_ bending modes at ∼1460 and ∼1374 cm^−1^ have also appeared from the hydrocarbon chains of OA and OLAm. Additionally, the spectrum of NPL solution shows a typical vibrational signature of the carboxylic acid group (*ν*_C=O_) at 1722 cm^−1^ along with the *ν*_N–H_ stretching mode at ∼3300 cm^−1^ from the amine moiety. The peak broadening of N–H stretching mode indicates the successful coordination of OLAm to the surface of nanostructures (Fig. S3, ESI†).^18^

**Fig. 2:**
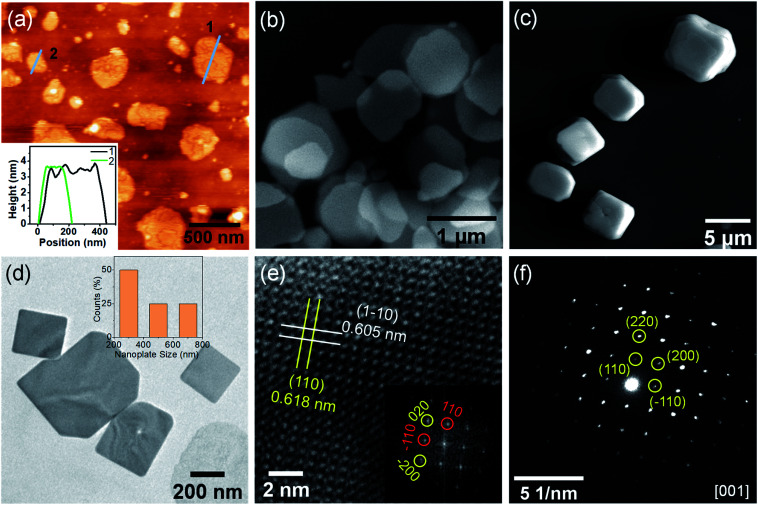
(a) AFM image of the RbPb_2_Br_5_ nanoplates (NPLs) synthesized by the LARP method. Inset: corresponding height profiles obtained from the image along the cyan lines. SEM images of RbPb_2_Br_5_ obtained by (b) LARP method and (c) mechanochemical grinding. (d) TEM image of the NPLs. Inset: size distribution histogram of NPLs. (e) HRTEM image of the NPLs exhibiting a deviation between interatomic distances along (110) and (1–10) directions. Inset: Fast Fourier Transformation (FFT) of the HRTEM image. (f) SAED pattern of the NPL.

The thickness of the NPLs was measured by atomic force microscopy (AFM) and the height profile measurements reveal a thickness of ∼3.5 nm of the NPLs (Fig. 2a), which corresponds to the 3–4 layers of RbPb_2_Br_5_. Further, the morphology of NPLs was explored by field emission scanning electron microscopy (FESEM), which further reveals thin 2D nanoplate morphology (Fig. 2b). The lateral dimension of NPLs ranges between ∼300–750 nm. The FESEM image of bulk RbPb_2_Br_5_ polycrystals shows the truncated cuboidal morphology with a particle size of 5–8 μm (Fig. 2c). Furthermore, the morphology of the LARP-synthesized sample without capping agent reveals a similar truncated cuboid with a particle size of 1.5–3 μm (Fig. S4, ESI†). Thereby, the results confirm that the capping ligands facilitate the directional growth of 2D ultrathin NPLs during LARP synthesis.

The transmission electron microscopy (TEM) image of the as-synthesized RbPb_2_Br_5_ obtained by the LARP method in the presence of OA and OLAm confirms the 2D NPL morphology (Fig. 2d), which is consistent with the directional growth revealed by the PXRD pattern (Fig. 1b). The particle size distribution histogram shows a broad size distribution of the NPLs (Fig. 2d, inset). High-resolution TEM (HRTEM) analysis reveals the single-crystalline nature of the NPLs (Fig. 2e). The tetragonal *I*4/*mcm* symmetry demands interplanar spacings for the (*hkl*) and (*h-kl*) family of lattice planes to be identical. Interestingly, the HRTEM analysis reveals that *d*_110_ and *d*_1–10_ are non-equivalent by 0.13 Å. The corresponding FFT analysis also exhibits a deviation of 0.11 Å between (110) and (−110) interplanar lattice spacings. Additionally, the (200) and (020) planes show a deviation of 0.08 Å in the interplanar distances (Fig. 2e, inset). Furthermore, we have carried out TEM analysis for bulk RbPb_2_Br_5_ (Fig. S5, ESI†) and found similar results. A careful inspection of the HRTEM image of the bulk sample reveals that the *d*_110_ and *d*_1–10_ are non-equivalent by 0.09 Å. The corresponding FFT analysis exhibits a deviation in (110) and (−110) interplanar lattice spacings by 0.07 Å (Fig. S5b, ESI†). These results show that the lattice parameters *a* and *b* are no longer equal on a local scale for NPLs and for bulk samples and signify the presence of intrinsic local distortion in the RbPb_2_Br_5_ lattice.^20^ This local distortion in the average tetragonal structure may give rise to the strong lattice anharmonicity,^21,22^ which will have an impact on the luminescence properties. The selected area electron diffraction (SAED) pattern of the RbPb_2_Br_5_ NPLs additionally confirms the single crystalline nature (Fig. 2f). The diffraction spots associated with the (110), (220) and (200) crystal planes can be indexed to the [001] zone axis of tetragonal RbPb_2_Br_5_.

**Fig. 3:**
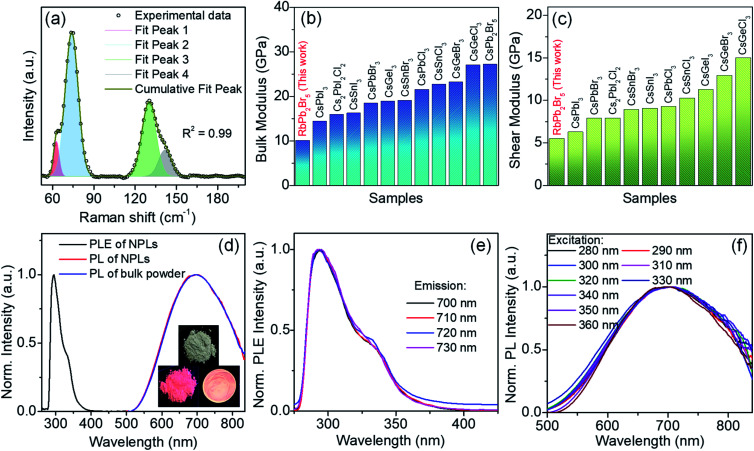
(a) Raman spectrum of RbPb_2_Br_5_ NPLs. Comparison of (b) bulk modulus and (c) shear modulus of RbPb_2_Br_5_ NPLs with several other all-inorganic metal halide perovskites such as CsPbX_3_ (X = Cl, Br, I),^26^ CsGeX_3_,^26^ CsSnX_3_ (X = Br, Cl),^26^ Cs_2_PbI_2_Cl_2_,^27^ CsSnI_3_,^28^ and CsPb_2_Br_5_.^29^ (d) PLE spectrum (black line) of RbPb_2_Br_5_ NPLs and PL spectra of NPLs (red line) and bulk polycrystals (blue line) of RbPb_2_Br_5_ at room temperature. Inset shows the images of bulk polycrystals under ambient light (top), UV light irradiation at 365 nm on bulk polycrystals and a drop cast film of NPLs (bottom). Wavelength-dependent (e) excitation and (f) emission spectra of RbPb_2_Br_5_ NPLs at room temperature.

To understand the lattice dynamics in 2D RbPb_2_Br_5_ NPLs, we have performed Raman spectroscopic investigation at room temperature. The distinctive vibrational modes appear from the PbBr_8_ polyhedron in RbPb_2_Br_5_ NPLs (Fig. 3a). Two strong Raman signals at ∼74 and 130 cm^−1^ are likely to emerge from the vibrations in the PbBr_8_ unit.^23^ Deconvolution of the peaks reveals the splitting of each peak into two components, which appear at 62.8, 73.6, 130.4 and 141.5 cm^−1^. This can be ascribed to the different types of Pb–Br bonding strength in the PbBr_8_ polyhedron corresponding to two different bond lengths, 3.15 Å (two longer bonds) and 2.87 Å (six shorter bonds), respectively.^24^ While the covalent bond distance between Pb and Br is about 2.66 Å, close to the sum of covalent radii of Pb (1.46 Å) and Br (1.20 Å),^25^ the Pb–Br ionic interaction distance is found to be 3.25 Å.^26^ The Rb–Br distance is 3.59 Å in RbPb_2_Br_5_^24^ which is close to the sum of the ionic radii of Rb^+^ (1.66 Å) and Br^−^ (1.96 Å).^26^ Thereby, RbPb_2_Br_5_ exhibits a chemical bonding hierarchy which induces anharmonicity and softness in the crystal lattice.

Quantitatively, the anharmonicity can be evaluated using the Grüneisen parameter (*γ*), which is defined as:^27^
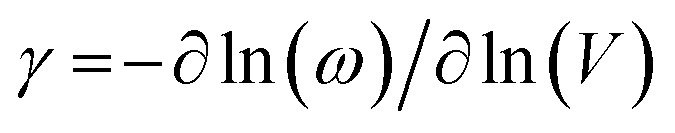
where *ω* is the frequency of the given phonon and *V* is the unit cell volume. *γ* indicates how the bond stiffness is affected by the change in the interatomic bond distances and the probability of phonon–phonon interaction is proportional to the square of *γ*. The value of *γ* is estimated to be 1.6 from the experimental measurement of the sound velocity of RbPb_2_Br_5_ NPLs (see methods in the ESI). The longitudinal (*v*_l_) and transverse sound velocity (*v*_t_) were measured to be 1744 m s^−1^ and 980 m s^−1^, respectively, which are reasonably low in comparison to other earlier reported metal halides (Table S1, ESI†).^28–31^ The average sound velocity (*v*_m_) was estimated using the formula:
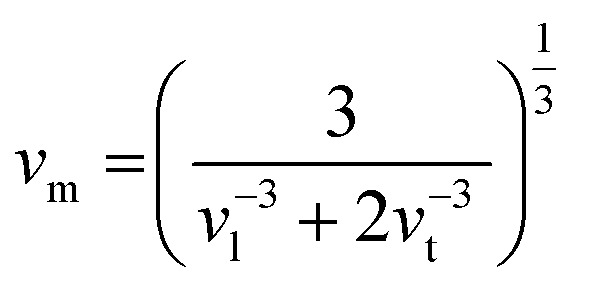
which exhibits a low value of 1090 m s^−1^. The high value of *γ* in RbPb_2_Br_5_ NPLs represents the presence of high lattice anharmonicity.^27^ The elastic moduli of the sample were estimated by using the following equations:
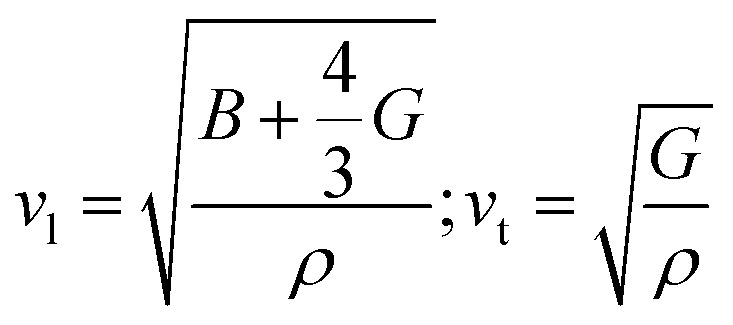
where *B* and *G* are the bulk and shear moduli respectively. *ρ* refers to the density of the sample. The estimated bulk and shear moduli (10.10 and 5.51 GPa, respectively) are found to be lower compared to other all-inorganic halide perovskites revealing a significantly softer lattice (Fig. 3b and c).^29,31–33^ The presence of lattice anharmonicity and a soft crystal structure is important for a system to show STE emission.^34^

The optical properties of the RbPb_2_Br_5_ bulk polycrystals and NPL solution in chloroform were investigated by electronic absorption and PL spectroscopy. Bulk RbPb_2_Br_5_ polycrystals show a band gap of 3.51 eV (Fig. S6a, ESI†). The absorption spectrum of RbPb_2_Br_5_ NPL solution shows both excitonic and band edge absorption features at 3.55 eV (350 nm) and 4.27 eV (290 nm), respectively, with a long absorption tail in the lower energy region (Fig. S6b, ESI†).^14,35,36^ The probable presence of sub-bandgap state transition might be responsible for the extended absorption tail in the lower energy region, which may have originated from the surface defects of the NPLs.^37,38^ The excitonic peak is a typical observation in 2D halide perovskites.^35^ The PLE spectrum of the NPLs exhibits a distinct peak at 294 nm corresponding to the absorption onset (Fig. 3d). At room temperature, a broadband emission ranging from ∼500 nm to the near infrared region (∼850 nm) is observed for both the NPL solution and bulk polycrystals centred at ∼695 nm, when excited at 370 nm (Fig. 3d). The broad emission centred at ∼695 nm is consistent with the pinkish white colour of both the bulk polycrystals and NPL thin film when irradiated with a 365 nm UV lamp (inset of Fig. 3d). Significant Stokes shifted PL (340–400 nm) with a large full width at half-maximum (FWHM) of 275 nm is observed for both RbPb_2_Br_5_ NPLs and bulk polycrystals (Fig. 3d, and S7, ESI†). The large Stokes shift of over 340 nm is desirable for light-emitting applications as it greatly reduces the self-absorption.^14,39^ In general, such broadband and highly Stokes shifted emission does not arise from direct band-to-band recombination. For instance, in alkali halides and organic–inorganic hybrid halide crystals the broadband emission is due to the strong electron–phonon interaction in the deformable lattice and associated self-trapped excitonic (STE) recombination.^14,34,40–43^ The large Stokes shift is caused by the energy loss in the process of excited state structural distortion.^34^

The as-synthesized RbPb_2_Br_5_ NPL sample shows an absolute PLQY of ∼13%, which is reasonably higher compared to that of the earlier reported broadband STE-emitting Pb based 2D halide perovskites (Table S2, ESI†). Similarly, few other all-inorganic STE-emitting halide materials, Cs_4_SnBr_6_ and Cs_2_AgBiCl_6_ exhibited PLQY in the range of ∼5 to 15%.^41,44^ The identical shape and features of the PLE spectra collected across the broad emission range as well as the excitation independent PL spectra indicate the broadband emission is originating from the relaxation of the same excited state (Fig. 3e and f and S7a and b, ESI†).^45^ Furthermore, the identical PL emissions from both bulk and NPL samples rule out the involvement of defect states, which also indicates the existence of STE states.

**Fig. 4:**
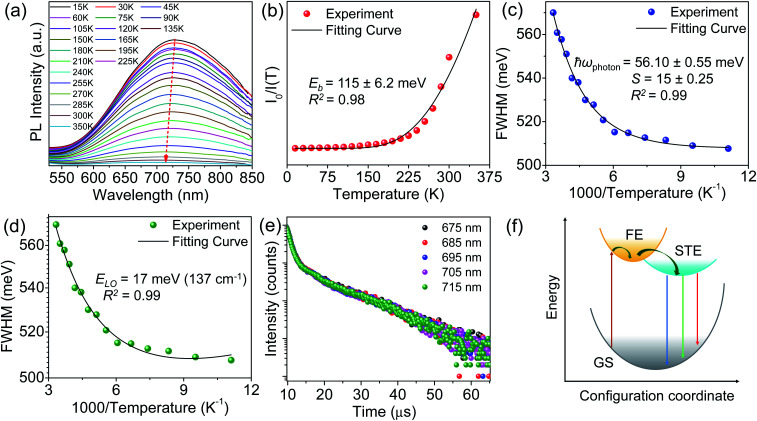
(a) Temperature-dependent PL spectra of RbPb_2_Br_5_ NPLs. (b) Arrhenius plot of integrated PL intensity *versus* temperature, with the solid black line corresponding to a least squared fit of the thermal quenching model. (c and d) Full width at half-maximum (FWHM) *versus* 1000/temperature, with least squared fits corresponding to phonon broadening following the Toyozaya model and Rudin's model portrayed by black lines, respectively. (e) Wavelength-dependent TRPL spectra of RbPb_2_Br_5_ NPLs at room temperature. (f) Configuration coordinate schematic diagram for the STE dynamic mechanism of RbPb_2_Br_5_ (GS = ground state, FE = free exciton state, and STE = self-trapped exciton state).

To probe the underlying mechanism of broadband emission in RbPb_2_Br_5_, temperature-dependent PL spectra were measured for the NPLs (Fig. 4a). Temperature-dependent PL spectra measured in the range of 15–350 K show a monotonous decrease in PL intensity and an increase in FWHM with increasing temperature indicating the increased probability of nonradiative relaxation and stronger electron–phonon coupling at higher temperature, respectively.^36,42,46^ Simultaneously, a slight blue shift in the emission peak is observed with the increase in temperature (Fig. 4a). Such a blueshift in PL for RbPb_2_Br_5_ NPLs can be ascribed to the slight shift in the relative positions of conduction and valence bands as temperature increases.^47,48^ No higher energy peak is observed at lower temperatures. Only one Gaussian-shaped broadband emission indicates that the self-trapping barrier is low, and the excitons are easily trapped.^43^ The thermal quenching behaviour is well fitted using the Arrhenius model,
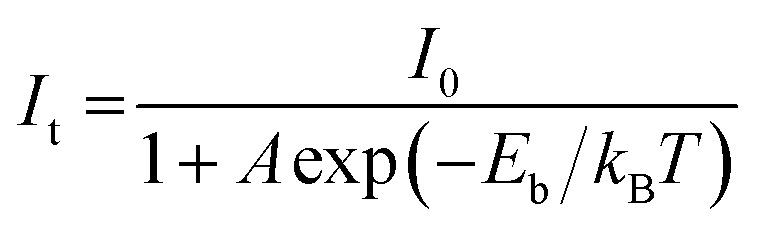
where *I*_0_ is the emission intensity at 0 K, *A* is the ratio between the radiative and the nonradiative decay rates, *k*_B_ is the Boltzmann constant and *E*_b_ is the exciton binding energy. The best fitted plot in Fig. 4b provides an *E*_b_ value of 115 ± 6.2 meV, which is much larger than that of the conventional 3D perovskite, CsPbX_3_ (∼18 meV), as well as thermal energy at room temperature (26 meV).^49^ This high *E*_b_ value is beneficial for radiative recombination.^50^ However, this value is lower compared to that of the 0D perovskites because of the weaker confinement.^51^

To get an estimation of the electron–phonon coupling in RbPb_2_Br_5_ NPLs, we further determined the Huang–Rhys electron–phonon coupling parameter (*S*) and effective phonon energy (*ħω*_phonon_) using the Toyozawa model,^42,52^
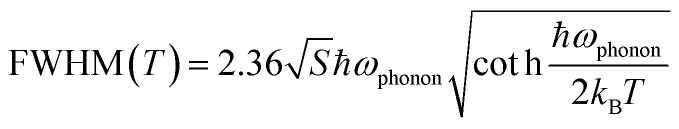
where *S* represents the distortion of the self-trapped state with respect to the ground state, *ħ* is the reduced Planck constant and *ω*_phonon_ is the phonon frequency. Through fitting of the experimental FWHM with the above model, we found an effective phonon energy of 56.10 meV with a high *S* of 15 (Fig. 4c). Similar large *S* values were also reported earlier for a few halides, such as Cs_2_AgBiBr_6_,^53^ Sb^3+^-doped Cs_2_NaInCl_6_,^54^ and (MA)_4_Cu_2_Br_6_,^55^ which showed STE emission due to strong electron–phonon coupling.

From the temperature dependent PL spectra, we can also evaluate the effective vibrational mode that couples with the electronic transition resulting in STE emission.^14^ Thereby, we fitted the FWHM to the Fröhlich longitudinal optical (LO) phonon broadening model developed by Rudin *et al.* (Fig. 4d) to extract the LO phonon energy using the following equation,^56,57^
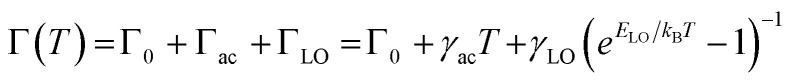
where *Γ*(*T*) is the PL line width and *Γ*_0_ originates due to the scattering of charge carriers from disorder which is temperature-independent and results in inhomogeneous broadening. *Γ*_ac_ and *Γ*_LO_ originate due to the scattering of charge carriers from the acoustic phonon and Fröhlich scattering of the longitudinal optical (LO) phonon, respectively, whereas, *γ*_ac_ and *γ*_LO_ are the corresponding coupling constants.^57^ From the fitting, we found the optical phonon energy (*E*_LO_) as 17 meV (137 cm^−1^) which is in good agreement with the Raman mode observed near 130 cm^−1^ in RbPb_2_Br_5_ NPLs (Fig. 3a). Thereby, the vibrational mode at ∼130 cm^−1^ is mainly responsible for the lattice deformation where electrons are trapped and leads to a broadband emission in 2D RbPb_2_Br_5_ NPLs. Interestingly, we found a high value of *γ*_LO_ of 431 meV which confirms a significant electron–phonon Fröhlich interaction within RbPb_2_Br_5_. This value is comparable to that of the other metal halide perovskites such as 0D Rb_3_BiCl_6_,^58^ 2D (F_2_CHCH_2_NH_3_)_2_Cd_*x*_Pb_1–*x*_Br_4_^59^ and Cs_3_Bi_2_I_6_Cl_3_,^60^ and also the 3D double perovskite Cs_2_AgBiBr_6_^53^ but much larger than that of the popular 3D lead halide perovskite, (FA/MA)Pb(I/Br)_3_^57^ (Table S3, ESI†). Thereby, the soft crystal lattice and high anharmonicity of RbPb_2_Br_5_ NPLs promote the lattice deformation due to strong electron–phonon interaction, which provides the STE and subsequent broadband PL emission.

Furthermore, we have carried out time-resolved PL (TRPL) measurements to gain more insight into the excitonic recombination dynamics in RbPb_2_Br_5_ NPLs. The PL decay curve is fitted using the double exponential function with an average lifetime (*τ*_avg_) of 10.03 μs (Fig. 4e and Table S4, ESI†). Additionally, we have performed the TRPL measurement of the NPL sample by monitoring across the wide emission range (675–715 nm), which shows a similar nature of decay kinetics (Fig. 4e) and infers an identical origin of broad emission. The bulk polycrystalline sample exhibited similar decay kinetics to the NPLs with an *τ*_avg_ of 8.82 μs (Fig. S8 and Table S4, ESI†). This observation suggests that the surface-to-bulk ratio has a minor impact on the PL decay kinetics.^61^ These results endorse the intrinsic characteristics of PL emission.^61^ Together with these findings, the assignment of the highly Stokes shifted broad PL emission in RbPb_2_Br_5_ can be ascribed to the phonon-assisted recombination of STEs. The excitation and recombination processes in RbPb_2_Br_5_ are schematically illustrated in Fig. 4f. Following the photon absorption, an electron is promoted to the excited state, and after its thermalization it gets self-trapped and localizes on Pb^2+^, which probably combines with another Pb^2+^ to form a quasi-molecule Pb_2_^3+^.^10,14^ Simultaneously, the hole may localize on Br^−^, which dimerizes with adjacent Br^−^ to form a molecular ion Br_2_^−^.^10,14^ This process leads to transient lattice distortion which is facilitated by a soft lattice and high anharmonicity. The self-trapped electron and hole then recombine together emitting broadband emission.

Finally, to achieve the band gap tuning in this material, the pure phase analogues of RbPb_2_Br_5_ were synthesized by the halide mixing strategy *via* mechanochemistry. The PXRD patterns of RbPb_2_Cl_2_Br_3_ and RbPb_2_Br_4_I reveal that both the materials possess similar tetragonal crystal structures to RbPb_2_Br_5_ (Fig. S9a, ESI†). The 2*θ* value shifts to a lower angle in RbPb_2_Br_4_I whereas it shifts to a higher angle in RbPb_2_Cl_2_Br_3_ in comparison to pure RbPb_2_Br_5_ because of the substitution of Br^−^ by larger I^−^ and smaller Cl^−^ ions, respectively (Fig. S9b, ESI†). The halide mixing effect is also reflected in optical spectroscopy. We have been able to tune the band gap in the 2.9–3.6 eV range by adjusting the halide composition in bulk RbPb_2_Br_5_. The band gap increases to 3.62 eV with Cl^−^ incorporation, while it decreases to 2.94 eV with I^−^ incorporation in comparison to the pure bromide system (3.51 eV) (Fig. S10, ESI†). The PL peak position also red shifts from RbPb_2_Cl_2_Br_3_ (∼685 nm) to RbPb_2_Br_5_ (∼695 nm) to RbPb_2_Br_4_I (∼765 nm) (Fig. S10b, SI†). Furthermore, we have synthesized the phase pure sample of the Cs-analogue (*i.e.*, CsPb_2_Br_5_) by mechanochemical grinding, as confirmed from its PXRD pattern (Fig. S11a, ESI†), which can also be indexed based on the tetragonal structure (space group *I*4/*mcm*).^15^ The solid-state electronic absorption spectrum of as synthesized CsPb_2_Br_5_ (Fig. S11b, ESI†) exhibits an absorption edge at ∼3.54 eV which is consistent with the previous reports.^9,62^ The PL spectrum of bulk CsPb_2_Br_5_ polycrystals (Fig. S11d, ESI†) revealed a narrow emission centred at 550 nm when excited at 350 and 380 nm.^62,63^ The narrow emission line for CsPb_2_Br_5_ in comparison to that for RbPb_2_Br_5_ might be ascribed to the suppression of electron–phonon coupling in CsPb_2_Br_5_.^64^ Thus, by changing only the A cation in between 2D [Pb_2_Br_5_]^−^ polyhedral units, the nature of PL emission varied immensely and subsequently, this exciting observation needs further detailed investigations.

## Conclusions

In summary, we have synthesized ultrathin NPLs of 2D layered RbPb_2_Br_5_ and controlled bulk polycrystals by simple ligand assisted reprecipitation and mechanochemical grinding, respectively. The NPLs display a highly Stokes shifted (∼400 nm) broadband PL emission with no overlap between their excitation and emission spectra. Furthermore, the NPLs exhibit a long lifetime component in the microsecond range. A soft crystal lattice along with high anharmonicity boosts structural distortion during photoexcitation where electrons can be easily trapped. As a result, 2D all-inorganic metal halide RbPb_2_Br_5_ exhibits strong electron–phonon coupling and STE with broadband emission. Furthermore, we have tuned the band gap in the range of ∼2.9–3.6 eV by adjusting the halide composition in the soft lattice of RbPb_2_Br_5_. Thus, our findings give a new direction towards the fundamental understanding of the emission process in low dimensional halides with the influence of chemical bonding, lattice anharmonicity and lattice dynamics which show potential prospects in a new generation of solid-state lighting and displays.

## Data availability

All data are available in the manuscript and in the ESI.†

## Author contributions

K. B. conceived the idea and designed the study. J. P., A. D., K. K. and K. B. carried out the synthesis, structural, optical, and sound velocity measurements, and other analyses. C. helped in the synthesis of NPLs. J. P. wrote the first draft; and A. D., K. K. and K. B. contributed to editing the manuscript.

## Conflicts of interest

The authors declare no conflict of interest.

## Supplementary Material

SC-013-D2SC02992H-s001

## References

[cit1] Stoumpos C. C., Kanatzidis M. G. (2015). Acc. Chem. Res..

[cit2] Pradhan N. (2019). ACS Energy Lett..

[cit3] Akkerman Q. A., Rainò G., Kovalenko M. V., Manna L. (2018). Nat. Mater..

[cit4] Dey A., Ye J., De A., Debroye E., Ha S. K., Bladt E., Kshirsagar A. S., Wang Z., Yin J., Wang Y., Quan L. N., Yan F., Gao M., Li X., Shamsi J., Debnath T., Cao M., Scheel M. A., Kumar S., Steele J. A., et a. (2021). ACS Nano.

[cit5] Protesescu L., Yakunin S., Bodnarchuk M. I., Krieg F., Caputo R., Hendon C. H., Yang R. X., Walsh A., Kovalenko M. V. (2015). Nano Lett..

[cit6] Swarnkar A., Chulliyil R., Ravi V. K., Irfanullah M., Chowdhury A., Nag A. (2015). Angew. Chem., Int. Ed..

[cit7] Wang K.-H., Wu L., Li L., Yao H.-B., Qian H.-S., Yu S.-H. (2016). Angew. Chem., Int. Ed..

[cit8] Ruan L., Shen W., Wang A., Xiang A., Deng Z. (2017). J. Phys. Chem. Lett..

[cit9] Acharyya P., Pal P., Samanta P. K., Sarkar A., Pati S. K., Biswas K. (2019). Nanoscale.

[cit10] Ogorodnikov I. N., Smirnov A. A., Pustovarov V. A., Isaenko L. I., Tarasova A. Y., Yakovlev V. Y. (2009). Phys. Solid State.

[cit11] Tarasova A. Y., Isaenko L. I., Kesler V. G., Pashkov V. M., Yelisseyev A. P., Denysyuk N. M., Khyzhun O. Y. (2012). J. Phys. Chem. Solids.

[cit12] Lavrentyev A. A., Gabrelian B. V., Vu V. T., Denysyuk N. M., Shkumat P. N., Tarasova A. Y., Isaenko L. I., Khyzhun O. Y. (2016). J. Phys. Chem. Solids.

[cit13] Yin J., Brédas J.-L., Bakr O. M., Mohammed O. F. (2020). Chem. Mater..

[cit14] Smith M. D., Karunadasa H. I. (2018). Acc. Chem. Res..

[cit15] Zhang Z., Zhu Y., Wang W., Zheng W., Lin R., Huang F. (2018). J. Mater. Chem. C.

[cit16] Li Z.-J., Hofman E., Li J., Davis A. H., Tung C.-H., Wu L.-Z., Zheng W. (2018). Adv. Funct. Mater..

[cit17] Li J., Xu L., Wang T., Song J., Chen J., Xue J., Dong Y., Cai B., Shan Q., Han B., Zeng H. (2017). Adv. Mater..

[cit18] Pradhan J., Moitra P., Umesh, Das B., Mondal P., Kumar G. S., Ghorai U. K., Acharya S., Bhattacharya S. (2020). Chem. Mater..

[cit19] Kundu K., Acharyya P., Maji K., Sasmal R., Agasti S. S., Biswas K. (2020). Angew. Chem., Int. Ed..

[cit20] Ghosh T., Samanta M., Vasdev A., Dolui K., Ghatak J., Das T., Sheet G., Biswas K. (2019). Nano Lett..

[cit21] Dutta M., Pal K., Etter M., Waghmare U. V., Biswas K. (2021). J. Am. Chem. Soc..

[cit22] Dutta M., Prasad M. V. D., Pandey J., Soni A., Waghmare U. V., Biswas K. (2022). Angew. Chem., Int. Ed..

[cit23] Rademaker K., Krupke W. F., Page R. H., Payne S. A., Petermann K., Huber G., Yelisseyev A. P., Isaenko L. I., Roy U. N., Burger A., Mandal K. C., Nitsch K. (2004). J. Opt. Soc. Am. B.

[cit24] Becker D., Beck H. P. (2004). Z. Anorg. Allg. Chem..

[cit25] Cordero B., Gómez V., Platero-Prats A. E., Revés M., Echeverría J., Cremades E., Barragán F., Alvarez S. (2008). Dalton Trans..

[cit26] Shannon R. D. (1976). Acta Crystallogr., Sect. A: Cryst. Phys., Diffr., Theor. Gen. Crystallogr..

[cit27] Dutta M., Sarkar D., Biswas K. (2021). Chem. Commun..

[cit28] Feng J. (2014). APL Mater..

[cit29] Xie H., Hao S., Bao J., Slade T. J., Snyder G. J., Wolverton C., Kanatzidis M. G. (2020). J. Am. Chem. Soc..

[cit30] Ferreira A. C., Létoublon A., Paofai S., Raymond S., Ecolivet C., Rufflé B., Cordier S., Katan C., Saidaminov M. I., Zhumekenov A. A., Bakr O. M., Even J., Bourges P. (2018). Phys. Rev. Lett..

[cit31] Acharyya P., Ghosh T., Pal K., Kundu K., Singh Rana K., Pandey J., Soni A., Waghmare U. V., Biswas K. (2020). J. Am. Chem. Soc..

[cit32] Roknuzzaman M., Ostrikov K., Wang H., Du A., Tesfamichael T. (2017). Sci. Rep..

[cit33] Ma Z., Li F., Qi G., Wang L., Liu C., Wang K., Xiao G., Zou B. (2019). Nanoscale.

[cit34] Li S., Luo J., Liu J., Tang J. (2019). J. Phys. Chem. Lett..

[cit35] Stoumpos C. C., Cao D. H., Clark D. J., Young J., Rondinelli J. M., Jang J. I., Hupp J.
T., Kanatzidis M. G. (2016). Chem. Mater..

[cit36] Dohner E. R., Jaffe A., Bradshaw L. R., Karunadasa H. I. (2014). J. Am. Chem. Soc..

[cit37] Yang B., Chen J., Hong F., Mao X., Zheng K., Yang S., Li Y., Pullerits T., Deng W., Han K. (2017). Angew. Chem., Int. Ed..

[cit38] Yang B., Chen J., Yang S., Hong F., Sun L., Han P., Pullerits T., Deng W., Han K. (2018). Angew. Chem., Int. Ed..

[cit39] Zhou L., Liao J.-F., Huang Z.-G., Wei J.-H., Wang X.-D., Li W.-G., Chen H.-Y., Kuang D.-B., Su C.-Y. (2019). Angew. Chem., Int. Ed..

[cit40] Williams R. T., Song K. S. (1990). J. Phys. Chem. Solids.

[cit41] Benin B. M., Dirin D. N., Morad V., Wörle M., Yakunin S., Rainò G., Nazarenko O., Fischer M., Infante I., Kovalenko M. V. (2018). Angew. Chem., Int. Ed..

[cit42] McCall K. M., Stoumpos C. C., Kostina S. S., Kanatzidis M. G., Wessels B. W. (2017). Chem. Mater..

[cit43] Lian L., Zheng M., Zhang P., Zheng Z., Du K., Lei W., Gao J., Niu G., Zhang D., Zhai T., Jin S., Tang J., Zhang X., Zhang J. (2020). Chem. Mater..

[cit44] Ke B., Zeng R., Zhao Z., Wei Q., Xue X., Bai K., Cai C., Zhou W., Xia Z., Zou B. (2020). J. Phys. Chem. Lett..

[cit45] Luo J., Wang X., Li S., Liu J., Guo Y., Niu G., Yao L., Fu Y., Gao L., Dong Q., Zhao C., Leng M., Ma F., Liang W., Wang L., Jin S., Han J., Zhang L., Etheridge J., Wang J., Yan Y., Sargent E. H., Tang J. (2018). Nature.

[cit46] Zhou L., Zhang L., Li H., Shen W., Li M., He R. (2021). Adv. Funct. Mater..

[cit47] Varshni Y. P. (1967). Physica.

[cit48] Frost J. M., Butler K. T., Brivio F., Hendon C. H., van Schilfgaarde M., Walsh A. (2014). Nano Lett..

[cit49] Yang H., Zhang Y., Pan J., Yin J., Bakr O. M., Mohammed O. F. (2017). Chem. Mater..

[cit50] Jing Y., Liu Y., Li M., Xia Z. (2021). Adv. Opt. Mater..

[cit51] Koutselas I. B., Ducasse L., Papavassiliou G. C. (1996). J. Phys.: Condens. Matter.

[cit52] Toyozawa Y. (1962). Prog. Theor. Phys..

[cit53] Steele J. A., Puech P., Keshavarz M., Yang R., Banerjee S., Debroye E., Kim C. W., Yuan H., Heo N. H., Vanacken J., Walsh A., Hofkens J., Roeffaers M. B. J. (2018). ACS Nano.

[cit54] Zeng R., Zhang L., Xue Y., Ke B., Zhao Z., Huang D., Wei Q., Zhou W., Zou B. (2020). J. Phys. Chem. Lett..

[cit55] Peng H., Yao S., Guo Y., Zhi R., Wang X., Ge F., Tian Y., Wang J., Zou B. (2020). J. Phys. Chem. Lett..

[cit56] Rudin S., Reinecke T. L., Segall B. (1990). Phys. Rev. B: Condens. Matter Mater. Phys..

[cit57] Wright A. D., Verdi C., Milot R. L., Eperon G. E., Perez-Osorio M. A., Snaith H. J., Giustino F., Johnston M. B., Herz L. M. (2016). Nat. Commun..

[cit58] Zhou L., Liao J.-F., Qin Y., Wang X.-D., Wei J.-H., Li M., Kuang D.-B., He R. (2021). Adv. Funct. Mater..

[cit59] Luo B., Liang D., Sun S., Xiao Y., Lian X., Li X., Li M.-D., Huang X.-C., Zhang J. Z. (2020). J. Phys. Chem. Lett..

[cit60] McCall K. M., Stoumpos C. C., Kontsevoi O. Y., Alexander G. C. B., Wessels B. W., Kanatzidis M. G. (2019). Chem. Mater..

[cit61] Thirumal K., Chong W. K., Xie W., Ganguly R., Muduli S. K., Sherburne M., Asta M., Mhaisalkar S., Sum T. C., Soo H. S., Mathews N. (2017). Chem. Mater..

[cit62] Shen P., Ma X., Pan F., Wang Y.-n., Liu B., Ye H. (2021). J. Phys. Chem. C.

[cit63] Pal P., Saha S., Banik A., Sarkar A., Biswas K. (2018). Chem.–Eur. J..

[cit64] Yang H., Shi W., Cai T., Hills-Kimball K., Liu Z., Dube L., Chen O. (2020). Nanoscale.

