# Supplementary material for: Soft crystal lattice and large anharmonicity facilitate the self-trapped excitonic emission in ultrathin 2D nanoplates of RbPb2Br5
Source: Chem Sci. 2022 Aug 1;13(34):9952–9. doi: 10.1039/d2sc02992h (PMC9430307; doi:10.1039/d2sc02992h)
Supplement: SC-013-D2SC02992H-s001 [file SC-013-D2SC02992H-s001.pdf]

Electronic Supplementary Material (ESI) for Chemical Science.
This journal is © The Royal Society of Chemistry 2022

Soft Crystal Lattice and Large Anharmonicity Facilitate the Self-Trapped Excitonic Emission in Ultrathin 2D Nanoplates of $RbPb_2Br_5$

Jayita Pradhan,[a] Anustoop Das,[a] Kaushik Kundu,[a] Chahat,[a] and Kanishka Biswas*[ab]

[a]New Chemistry Unit, [b]School of Advanced Materials and International Centre for Materials Science, Jawaharlal Nehru Centre for Advanced Scientific Research (JNCASR), Jakkur P.O., Bangalore 560064, India.

E-mail: kanishka@jncasr.ac.in

Experimental Methods

Materials. Rubidium bromide (RbBr, 99.60%, Sigma-Aldrich), lead (II) bromide ($PbBr_2$, 99.99%, Sigma-Aldrich), lead (II) chloride ($PbCl_2$, 99.99 %), lead (II) iodide (PbI_2, 99.99%) chloroform ($CHCl_3$, spectroscopic grade), oleylamine (OLAm, technical grade, 90%), oleic acid (OA, technical grade, 90%) and dimethyl sulfoxide (DMSO) were used without further purification.

Synthesis of $RbPb_2Br_5$ by ligand-assisted reprecipitation (LARP) method. In a typical synthesis, RbBr (0.1 mmol) and $PbBr_2$ (0.2 mmol) were dissolved in 3 mL DMSO in a 15 mL glass vial to form a clear precursor solution. Then 5 mL chloroform was taken in another 15 mL vial and 500 μL of precursor solution was added rapidly into chloroform under vigorous stirring. The crude solution was centrifuged for 5 min at 6000 rpm, and the upper part of solution was discarded after centrifugation. The precipitate was redispersed in chloroform and again centrifuged at 6000 rpm for 5 min. Finally, the precipitate was collected as a final product and used for further characterization. In the ligand-assisted reaction, 0.5 mL OA and 30 μL OLAm were added to the precursor solution and stirred before injection into 5 mL of chloroform. Next steps were similar to the above-mentioned process.

Synthesis of $RbPb_2Br_5$, $RbPb_2Br_4I$ and $RbPb_2Cl_2Br_3$ by solid state mechanochemical grinding. In a typical synthesis of bulk powder of $RbPb_2Br_5$, 183.9 mg RbBr and 816.1 mg $PbBr_2$ were taken in a mortar pestle and grounded mechanically for 2 hr. To synthesize $RbPb_2Br_4I$, 224.4 mg RbI and 775.6 mg $PbBr_2$ were grounded mechanically in mortar pestle for 2 hr. $RbPb_2Cl_2Br_3$ was synthesized by grinding of 204 mg RbBr, 452.8 mg $PbBr_2$ and 343.1 mg $PbCl_2$ in a mortar pestle for 2 hr. All grindings were performed in an ambient condition.

Powder X-ray diffraction (PXRD). Rigaku Smart Lab diffractometer was used to collect PXRD patterns at room temperature using Cu Kα radiation (λ =1.5406 Å) with accelerating voltage of 40 kV (current of 30 mA).

Thermogravimetric analysis (TGA). Thermogravimetric analysis was carried out using a 2 STAR TGA instrument, in which the samples were heated in N_2 atmosphere (40 mL min^{-1}) at a rate of 5 °C min^{-1} in the temperature range of 25−900 °C.

Fourier transform infrared spectroscopy (FTIR). FTIR spectra were recorded in the range of 400-4000 cm^{-1} by using a Bruker Optics Alpha-P FTIR spectrophotometer equipped with an attenuated total reflectance (ATR) module.

Transmission electron microscopy (TEM). The TEM measurement was performed using a JEOL (JEM3010) TEM instrument (200 kV accelerating voltage) fitted with a Gatan CCD camera.

Atomic force microscopy (AFM). AFM imaging was carried out using Asylum Research MFP-3D AFM in tapping mode using AC160TS silicon probes, with nominal tip radii <10 nm.

Field emission scanning electron microscopy (FESEM). FESEM images were acquired by a ZEISS Gemini SEM – Field Emission Scanning Electron Microscopy.

Raman spectroscopy. Raman spectroscopy measurements of the powder sample were carried out with a Renishaw spectrometer. The excitation wavelength of the laser was 532 nm.

Sound velocity measurement. The longitudinal (v_l) and transverse (v_t) sound velocities were measured on a disc shaped sample by using an Epoch 650 Ultrasonic Flaw Detector (Olympus) instrument with the transducer frequency of 5 MHz.

The Poisson's ratio (v_p) is linked with the sound velocities by this following equation,

$$v_p = \frac{1 - 2(\frac{v_t}{v_l})^2}{2 - 2(\frac{v_t}{v_l})^2}$$

The Grüneisen parameter (γ) was calculated using the following equation,

$$\gamma = \frac{3}{2}.\frac{1 + v_p}{2 - 3v_p}$$

Optical spectroscopy. Electronic absorption spectroscopy of $RbPb_2Br_5$ nanoplates (NPLs) solution in $CHCl_3$ was performed by PerkinElmer, Lambda-900 UV/vis/near-IR spectrometer. Diffuse reflectance measurements were carried out to estimate the optical band gap for solid samples in the range of 250−800 nm by using a Perkin-Elmer Lambda 900 UV/Vis/near-IR spectrometer in reflectance mode. By using the Kubelka-Munk equation, absorption (α/S) data was calculated from reflectance data: $\alpha/S = (1-R)^2/(2R)$, where R is the reflectance, α and S are the absorption and scattering coefficients respectively. The band gap for solid sample were derived from α/S vs. E (eV) plots.

All the photoluminescence measurements of the $RbPb_2Br_5$ NPLs solution in $CHCl_3$ and solid samples were recorded on an Edinburgh FLS1000 spectrofluorometer. Temperature-

dependent PL measurements ranging from 15 to 350 K were carried out using a vacuum cryostat. The TRPL decays of $RbPb_2Br_5$ NPL and bulk polycrystal were recorded at 370 nm excitation on Edinburgh FLS1000 spectrofluorometer instrument coupled with pulsed Xenon microsecond flash-lamp. The bi-exponential PL decay curves can be represented according to the following equation,

$$A(t) = A_1 e^{(-t/\tau_1)} + A_2 e^{(-t/\tau_2)}$$

where, A is the intensity of the emission at given time t. A_1 and A_2 are fitting parameters (i.e., relative amplitude for the different lifetime component), and $\tau_1$and τ_2 represent different lifetime components. The average lifetime ($\tau_{avg.}$) values of NPLs were calculated by following equation:

$$\tau_{avg.} = \frac{A_1 \tau_1^2 + A_2 \tau_2^2}{A_1 \tau_1 + A_2 \tau_2}$$

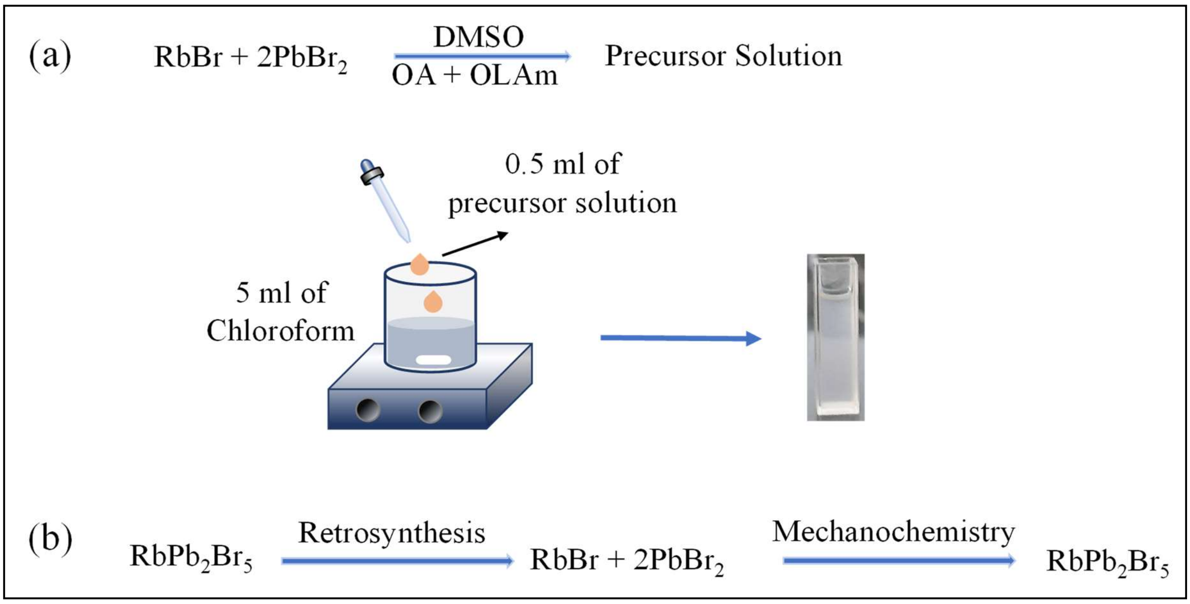

Scheme S1. Synthesis scheme of $RbPb_2Br_5$ *via* (a) ligand-assisted re-precipitation (LARP) method, and (b) solid state mechanochemical grinding approach.

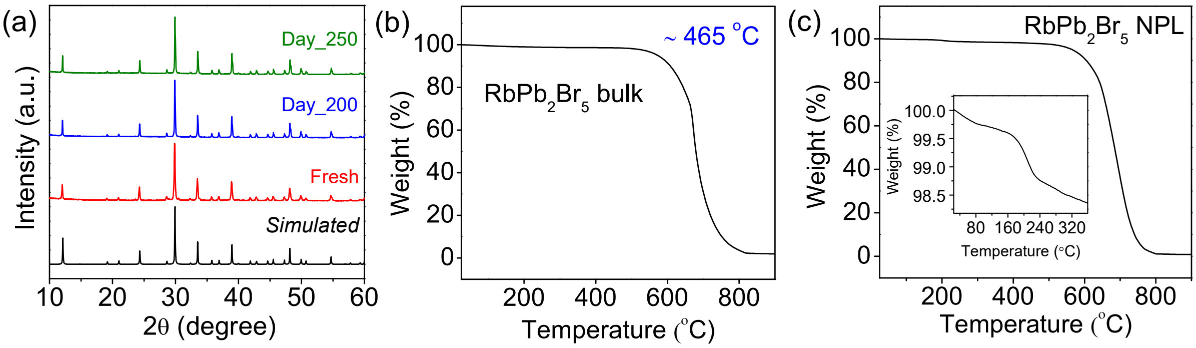

Fig. S1. (a) PXRD patterns of as-synthesized $RbPb_2Br_5$ bulk powder and after keeping in ambient conditions for 200 and 250 days. Thermogravimetric analysis (TGA) of (b) bulk $RbPb_2Br_5$ polycrystals and (c) NPLs. Inset in (c) shows zoomed lower temperature region.

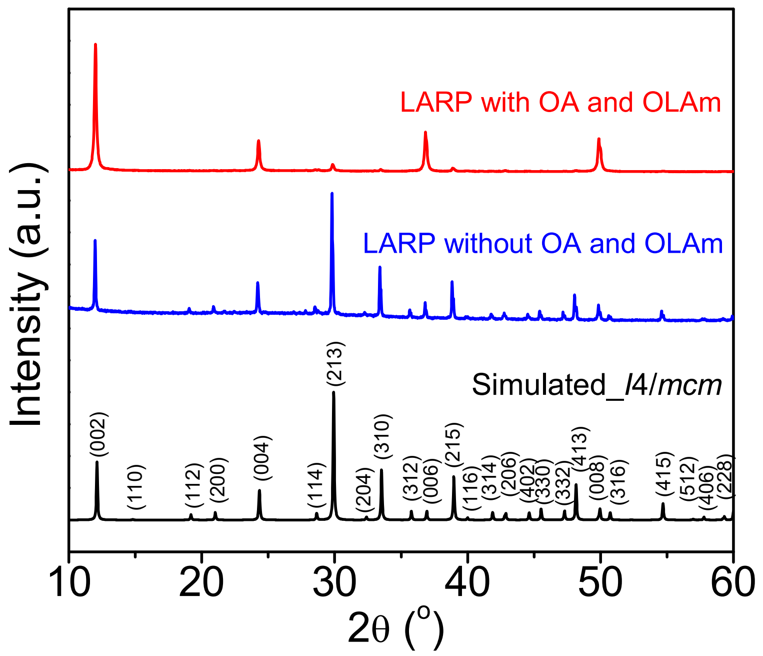

Fig. S2. PXRD patterns of $RbPb_2Br_5$ synthesized by LARP method with and without the addition of OA and OLAm.

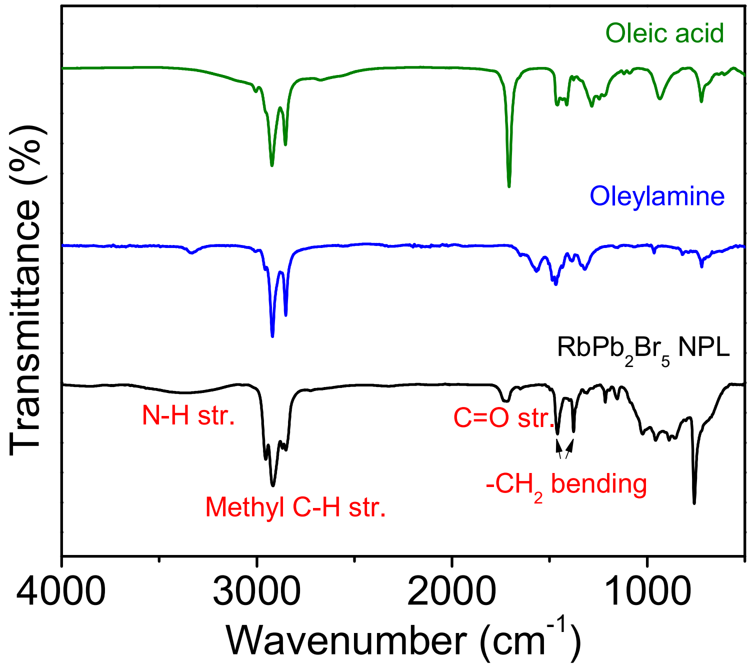

Fig. S3. FTIR spectra of $RbPb_2Br_5$ nanoplates (NPLs); and for pure OLAm and OA.

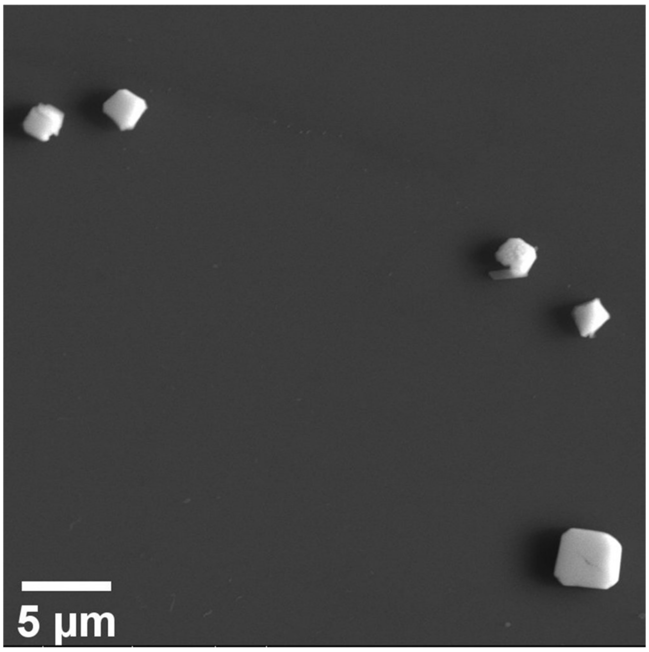

Fig. S4. FESEM image of $RbPb_2Br_5$ obtained *via* LARP method without the addition of OA and OLAm.

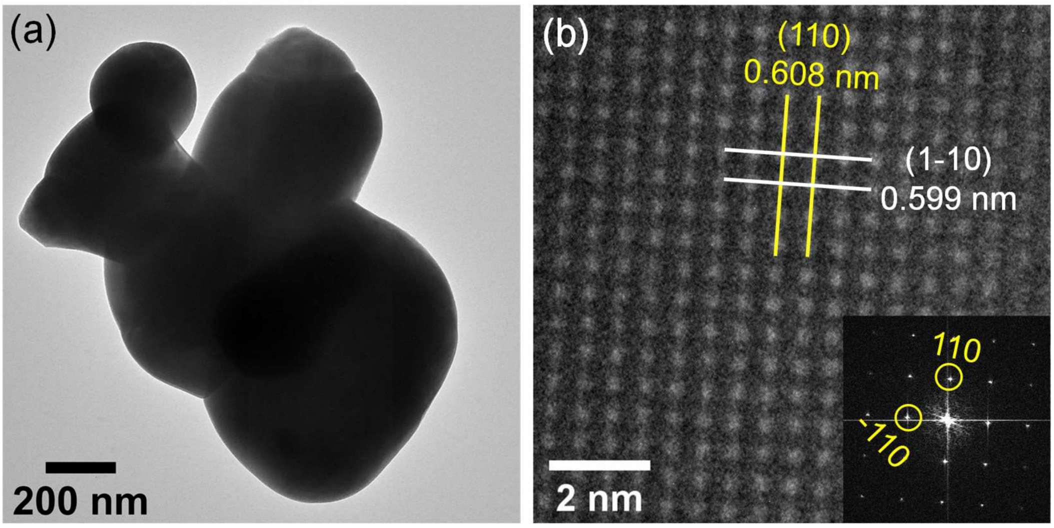

Fig. S5. (a) TEM image of $RbPb_2Br_5$ bulk polycrystal. (b) HRTEM image of the bulk polycrystal exhibiting slight deviation between interplanar distances along (110) and (1-10) directions. Inset: Fast Fourier transformation (FFT) of the HRTEM image.

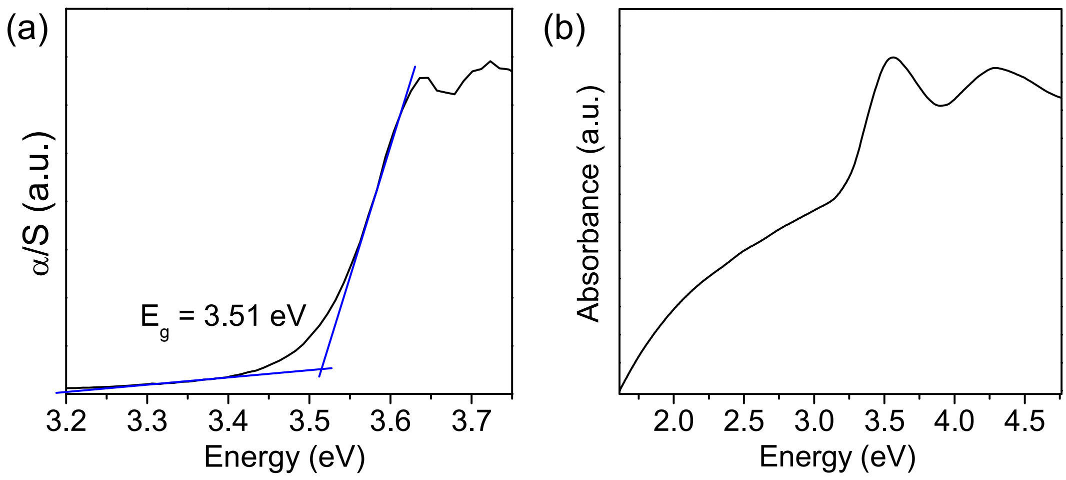

Fig. S6. (a) Solid-state electronic absorption of bulk $RbPb_2Br_5$ polycrystals. (b) Optical absorption spectrum of $RbPb_2Br_5$ NPLs in solution.

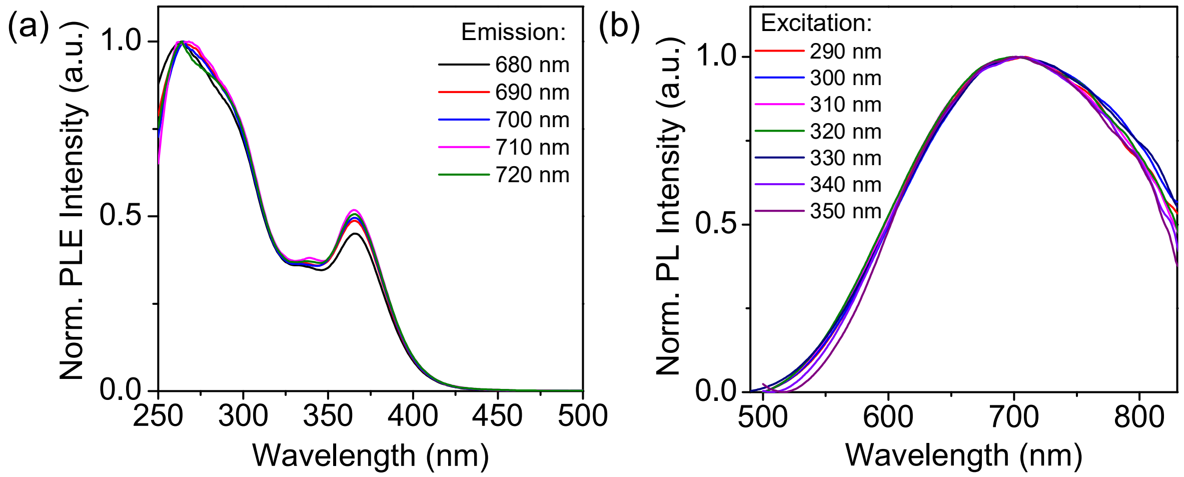

Fig. S7. Wavelength-dependent (a) excitation and (b) emission spectra of bulk $RbPb_2Br_5$ polycrystals at room temperature.

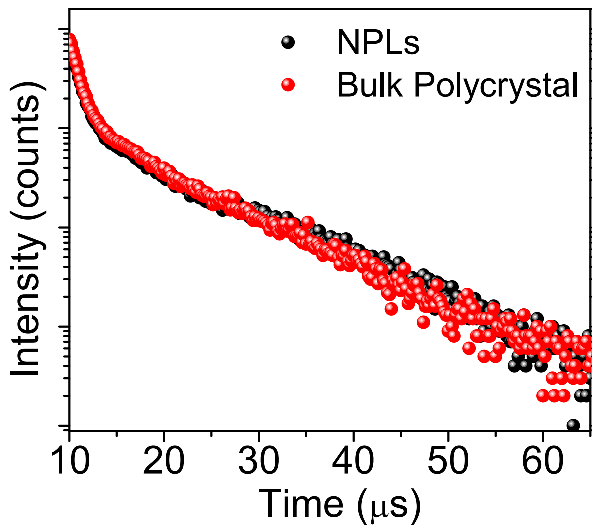

Fig. S8. TRPL spectra for $RbPb_2Br_5$ NPLs and bulk polycrystal at room temperature.

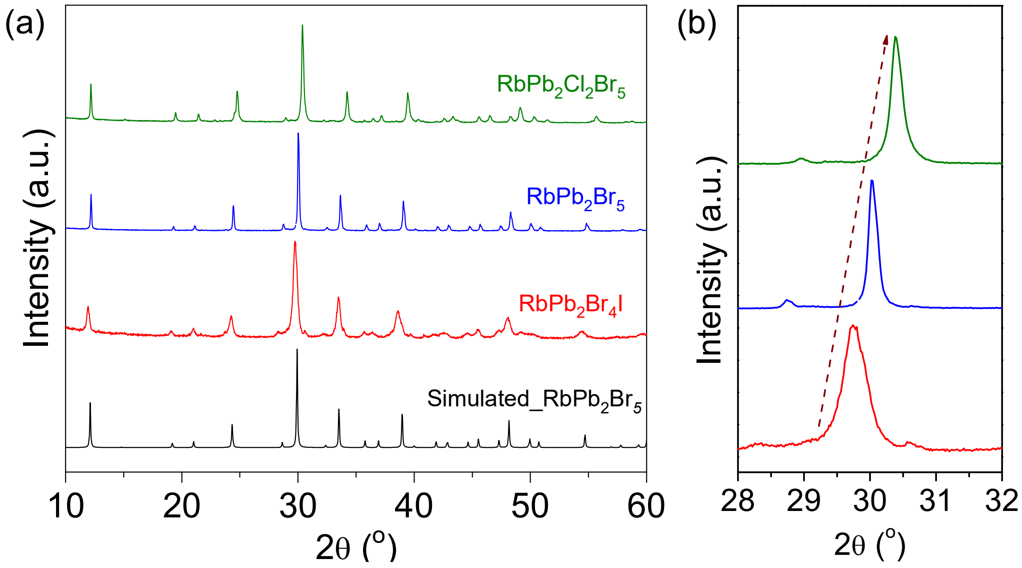

Fig. S9. (a) PXRD patterns along with the (b) peak shifting of $RbPb_2Br_5$, $RbPb_2Br_4I$, and $RbPb_2Cl_2Br_3$ bulk polycrystals.

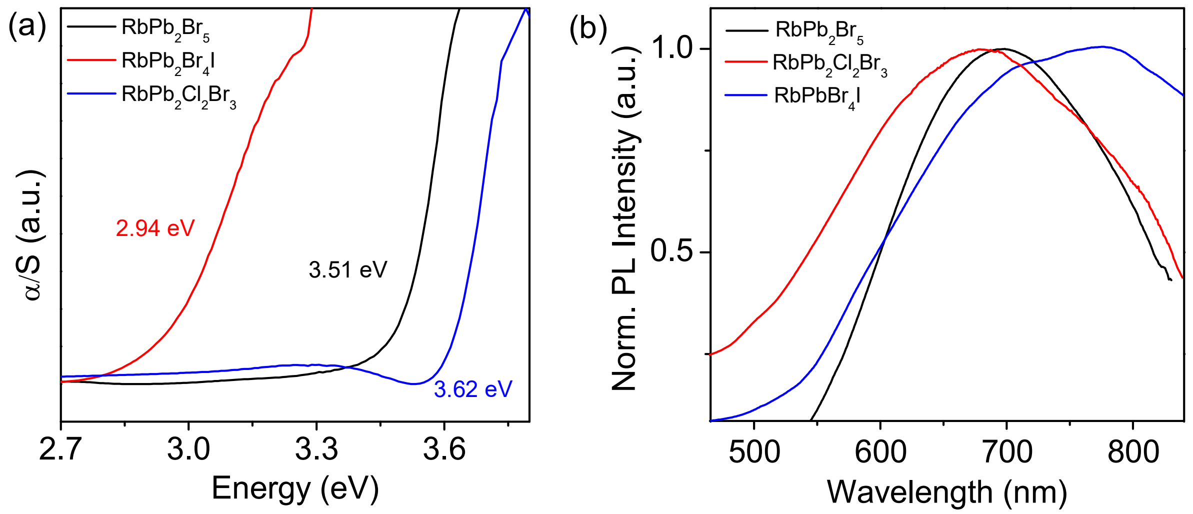

Fig. S10. (a) Solid-state electronic absorption (α/S) spectra and (b) emission spectra of $RbPb_2Br_5$, $RbPb_2Br_4I$, and $RbPb_2Cl_2Br_3$ bulk polycrystals at room temperature.

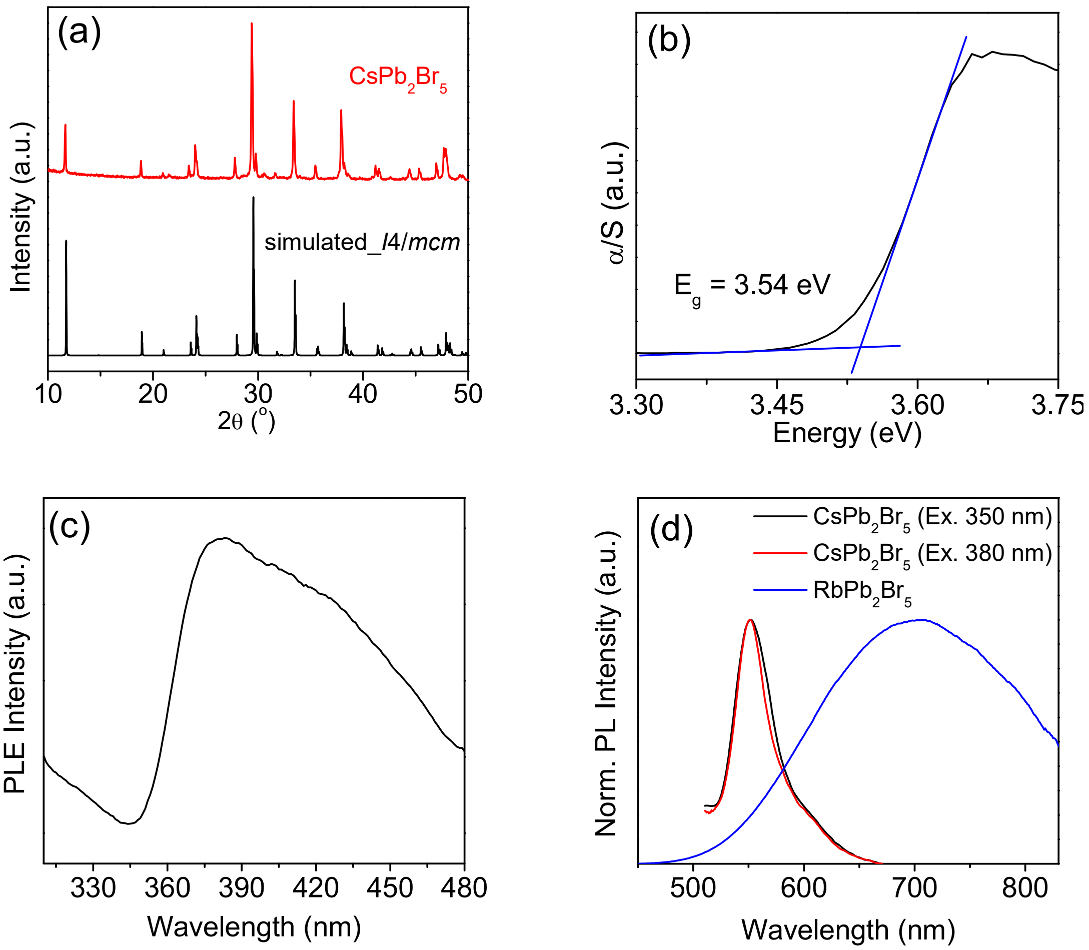

Fig. S11. (a) PXRD pattern, (b) solid-state electronic absorption spectrum (α/S) of $CsPb_2Br_5$. (c) Photoluminescence excitation (PLE) spectrum. (d) Comparison of the PL spectra of $CsPb_2Br_5$ and $RbPb_2Br_5$ bulk polycrystal at room temperature.

Table S1. Comparison of measured sound velocity of present $RbPb_2Br_5$ sample with that of reported values other metal halides.

Material	Longitudinal sound velocity (v_l) (m/s)	Transverse sound velocity (v_t) (m/s)	Method	References
$MASnI_3$	2469-3177	1249-1855	Theoretical	Ref. 1
$MASnBr_3$	3172-3605	1584-2043	Theoretical	Ref. 1
$CsSnI_3$	1549	967	Experimental	Ref. 2
$CsSnBr_3$	2422	1141	Experimental	Ref. 2
$FAPbI_3$	1650 ± 150	800 ± 50	INS (Exp)	Ref. 3
$FAPbBr_3$	2700 ± 80	900-1400	INS (Exp)	Ref. 3
$MAPbI_3$	2300 ± 70	1330 ± 30	INS (Exp)	Ref. 3
$MAPbBr_3$	3000 ± 50	1130-1320	INS (Exp)	Ref. 3
$Cs_2PbI_2Cl_2$	2460	1360	Theoretical	Ref. 4
**$RbPb_2Br_5$**	**1744**	**980**	**Experimental**	**This work**

Table S2. Comparison of reported PLQY of other 2D lead halide perovskites with that of present work.

Material	Dimensionality	PLQY	References
$(4NPEA)_2PbI_4$ (4NPEA = 4-nitrophenylethylammonium)	2D	(0.4 ± 0.1)%	Ref. 5
$(EDBE)PbBr_4$ (EDBE = 2,2′-(ethylenedioxy)bis(ethylammonium))	2D	9%	Ref. 6
PEA_2PbI_4 (PEA = phenylethylammonium)	2D	0.7%	Ref. 7
$(N\text{-}MEDA)[PbBr_4]$ (N-MEDA =*N*[1]-methylethane-1,2-diammonium)	2D	~0.5%	Ref. 8
$(NMEDA)[PbBr_{2.8}Cl_{1.2}]$	2D	1.5 %	Ref. 8
**$RbPb_2Br_5$**	**2D**	**13%**	**This work**

Table S3. Comparison of reported electron-longitudinal optical phonon coupling constant for other halides with that of present work.

Material	γ_{LO} (meV)	Dimensionality	References
Rb_3BiCl_6	693	0D	Ref. 9
Rb_3BiCl_6:4.5%Sb	347	0D	Ref. 9
$(C_4H_{14}N_2)_2In_2Br_{10}$	178	0D	Ref. 10
$(C_6H_8N_3)_2Pb_2Br_{10}\cdot H_2O$	150	1D	Ref. 11
$(F_2CHCH_2NH_3)_2Cd_xPb_{1-x}Br_4$	467	2D	Ref. 12
$Cs_3Bi_2I_6Cl_3$	291.8	2D	Ref. 13
$(C_6H_5C_2H_4NH_3)_2PbCl_4$	265	2D	Ref. 14
$(PEA)_2(CsPbBr_3)_{n-1}PbBr_4$	204-302	2D	Ref. 15
$Cs_2AgBiBr_6$	226	3D	Ref. 16
$FAPbI_3$	40	3D	Ref. 17
$FAPbBr_3$	61	3D	Ref. 17
$MAPbI_3$	40	3D	Ref. 17
$MAPbBr_3$	58	3D	Ref. 17
**$RbPb_2Br_5$**	**431**	**2D**	**This work**

Table S4. Fitted lifetime values using a bi-exponential decay with the relative amplitude of each component and average lifetime of $RbPb_2Br_5$ NPL and bulk polycrystal at room temperature.

Sample	τ_1 (μs)	τ_2 (μs)	A_1(%)	A_2(%)	$\tau_{avg.}$ (μs)
Nanoplate	1.47	10.25	15.01	84.99	10.03
Bulk Polycrystal	1.33	8.99	12.98	87.02	8.82

References

1 J. Feng, *APL Mater.*, 2014, **2**, 081801.

2 H. Xie, S. Hao, J. Bao, T. J. Slade, G. J. Snyder, C. Wolverton and M. G. Kanatzidis, *J. Am. Chem. Soc.*, 2020, **142**, 9553–9563.

3 A. Ferreira, A. Létoublon, S. Paofai, S. Raymond, C. Ecolivet, B. Rufflé, S. Cordier, C. Katan, M. I. Saidaminov and A. Zhumekenov, *Phys. Rev. Lett.*, 2018, **121**, 085502.

4 P. Acharyya, T. Ghosh, K. Pal, K. Kundu, K. Singh Rana, J. Pandey, A. Soni, U. V. Waghmare and K. Biswas, *J. Am. Chem. Soc.*, 2020, **142**, 15595−15603.

5 M. H. Tremblay, F. Thouin, J. Leisen, J. Bacsa, A. R. Srimath Kandada, J. M. Hoffman, M. G. Kanatzidis, A. D. Mohite, C. Silva, S. Barlow and S. R. Marder, *J. Am. Chem. Soc.*, 2019, **141**, 4521−4525.

6 J. E. Thomaz, K. P. Lindquist, H. I. Karunadasa and M. D. Fayer, *J. Am. Chem. Soc.*, 2020, **39**, 16622–16631.

7 J. Yu, J. Kong, W. Hao, X. Guo, H. He, W. R. Leow, Z. Liu, P. Cai, G. Qian, S. Li, X. Chen and X. Chen, *Adv. Mater.*, 2019, **31**, e1806385.

8 E. R. Dohner, E. T. Hoke and H. I. Karunadasa, *J. Am. Chem. Soc.*, 2014, **136**, 1718–1721.

9 L. Zhou, J.-F. Liao, Y. Qin, X.-D. Wang, J.-H. Wei, M. Li, D.-B. Kuang and R. He, *Adv. Funct. Mater.*, 2021, **31**, 2102654.

10 L. Zhou, J.-F. Liao, Z.-G. Huang, J.-H. Wei, X.-D. Wang, H.-Y. Chen and D.-B. Kuang, *Angew. Chem., Int. Ed.*, 2019, **58**, 15435−15440.

11 A. Biswas, R. Bakthavatsalam, S. R. Shaikh, A. Shinde, A. Lohar, S. Jena, R. G. Gonnade and J. Kundu, *Chem. Mater.*, 2019, **31**, 2253−2257.

12 B. Luo, D. Liang, S. Sun, Y. Xiao, X. Lian, X. Li, M.-D. Li, X.-C. Huang and J. Z. Zhang, *J. Phys. Chem. Lett.*, 2020, **11**, 199−205.

13 K. M. McCall, C. C. Stoumpos, O. Y. Kontsevoi, G. C. B. Alexander, B. W. Wessels and M. G. Kanatzidis, *Chem. Mater.*, 2019, **31**, 2644−2650.

14 K. Thirumal, W. K. Chong, W. Xie, R. Ganguly, S. K. Muduli, M. Sherburne, M. Asta, S. Mhaisalkar, T. C. Sum, H. S. Soo and N. Mathews, *Chem. Mater.*, 2017, **29**, 3947−3953.

15 H. Long, X. Peng, J. Lu, K. Lin, L.-Q. Xie, B.-P. Zhang, L. Ying and Z. Wei, *Nanoscale*, 2019, **11**, 21867−21871.

16 W. Kim, H. Yuan, N. H. Heo, J. Vanacken, A. Walsh, J. Hofkens and M. B. J. Roeffaers, *ACS Nano*, 2018, **12**, 8081−8090.
17 A. D. Wright, C. Verdi, R. L. Milot, G. E. Eperon, M. A. Pérez-Osorio, H. J. Snaith, F. Giustino, M. B. Johnston and L. M. Herz, *Nat. Commun.*, 2016, **7**, 11755.
